# Assessment on the use of allopurinol to improve safety and efficacy of mercaptopurine in pediatric patients with Acute Lymphoblastic Leukemia and Lymphoma during maintenance therapy

**DOI:** 10.1002/cnr2.1987

**Published:** 2024-02-13

**Authors:** Tecca Barone, Smita Dandekar, Daniel McKeone, Kevin Mulieri

**Affiliations:** ^1^ Department of Pharmacy Penn State Milton S. Hershey Medical Center Hershey Pennsylvania USA; ^2^ Division of Pediatric Hematology/Oncology Penn State Children's Hospital Hershey Pennsylvania USA

**Keywords:** absolute neutrophil count, acute lymphoblastic leukemia and lymphoma, allopurinol, hepatotoxicity, mercaptopurine

## Abstract

**Background:**

Mercaptopurine is an important component of acute lymphoblastic leukemia (ALL) and lymphoma (LLy) maintenance therapy. The 6‐thioguanine nucleosides (6‐TGN) are believed to be the primary contributor to myelosuppression and immunosuppressive effects, while 6‐methylmercaptopurine (6‐MMPN) is believed to be responsible for several toxicities including hepatotoxicity, pancreatitis, and hypoglycemia. Previous reports suggest the addition of allopurinol may reduce these toxicities.

**Aims:**

To assess the use of allopurinol to improve both safety and efficacy of mercaptopurine in pediatric patients with ALL and LLy during maintenance therapy. Secondary objectives included evaluating patient tolerability and skewed metabolism. In addition, we also analyzed mercaptopurine daily dose reduction upon allopurinol initiation.

**Methods and Results:**

The primary endpoint was time within goal ANC prior to and after initiation of allopurinol. Secondary endpoints included; improvement in selective toxicities (hepatotoxicity, pancreatitis, and hypoglycemia) and 6‐MMPN to 6‐TGN ratio prior to and after allopurinol initiation. In addition, an exploratory endpoint assessing mercaptopurine daily dose reduction prior to and after allopurinol initiation was included. Sixteen patients met inclusion criteria and 15 (94%) of which were included in this study. Median percent of maintenance days within goal ANC prior to and after initiation of allopurinol was 27.8 (IQR 22.6–44.9) and 41.6 (IQR 20.2–58.2) respectively. All patients experienced selective toxicities; 15 (100%) hepatotoxicity, 1 (7%) pancreatitis, and 3 (20%) hypoglycemia. Improvement of toxicities was seen in 13/15 (87%), 1/1 (100%), and 2/3 (67%) respectively. Average 6‐MMPN:6‐TGN ratio prior to allopurinol initiation was 304:1 and after, allopurinol initiation improved to 15:1, resulting in a 95% reduction. Average mercaptopurine dose prior to and after allopurinol initiation decreased by about 56% (63 to 28 mg/m^2^/day).

**Conclusion:**

Results suggest that the use of allopurinol in pediatric patients with ALL and LLy receiving mercaptopurine during maintenance therapy is both safe and effective.

## 1 INTRODUCTION

Acute lymphoblastic leukemia (ALL) is a commonly diagnosed pediatric cancer and has a high overall 5‐year survival rate of about 90%.^1^ An important component of treatment is maintenance therapy.^2^ Despite high success rates, metabolites of mercaptopurine may lead to toxicities that result in an interruption of maintenance therapy. While a majority of these toxicities are reversible, interruption of therapy and reduced dose intensity may result in inferior patient outcomes.^3^

Mercaptopurine is a purine antagonist that leads to immunosuppression and produces anti‐leukemic effects. The 6‐thioguanine nucleosides (6‐TGN) metabolites are believed to be the primary contributor to myelosuppression and immunosuppressive effects, while the 6‐methylmercaptopurine (6‐MMPN) metabolite is believed to be responsible for several toxicities including hepatotoxicity, pancreatitis, and hypoglycemia.^4^ In addition, xanthine oxidase (XO) metabolism inactivates up to 70% of mercaptopurine resulting in poor bioavailability.^5^

Allopurinol inhibits XO, increasing the bioavailability of mercaptopurine.^5^ It is hypothesized that this shunts mercaptopurine metabolism toward 6‐TGN metabolites. Case series have been published on the use of allopurinol in combination with mercaptopurine for treatment of irritable bowel disease and leukemia. These studies suggested that addition of allopurinol may decrease toxicities and optimize absolute neutrophil count (ANC) making it a viable addition to maintenance regimens.^6^, ^7^, ^8^, ^9^, ^10^ Due to the bioavailability increase, mercaptopurine is empirically dose reduced by at least 50% upon allopurinol initiation. In summary, allopurinol has been added to mercaptopurine maintenance therapy regimens to abate toxicities and increase antileukemic effects measured by ANC.

The limited data published on this strategy has focused on reduction in hepatotoxicity. Here we report Penn State Health Children's Hospital's experience to decrease mercaptopurine toxicity while optimizing mercaptopurine myelosuppression. The primary objective of this retrospective review was to assess the use of allopurinol to improve both safety and efficacy of mercaptopurine in pediatric patients with ALL and LLy during maintenance therapy. Secondary objectives included evaluating patient tolerability and skewed metabolism. In addition, we also analyzed mercaptopurine daily dose reduction upon allopurinol initiation.

## 2 METHODS

This is a single‐center retrospective chart review report assessing safety and efficacy of mercaptopurine in pediatric patients with ALL and LLy during maintenance therapy at Penn State Health Children's Hospital (Hershey, PA, USA). This is a 146‐bed pediatric hospital with 18 dedicated hematology/oncology beds.^11^ Patients treated by the pediatric hematology/oncology service with a diagnosis of ALL or LLy that utilized allopurinol during maintenance therapy between November 12, 2011 and August 31, 2022 were assessed for inclusion. Exclusion was only achieved in the case of incomplete documentation. The study was approved by the Penn State Institutional Review Board.

Providers had the option to add allopurinol to maintenance regimens in patients suspected to have toxicities secondary to mercaptopurine at any point during maintenance therapy. Allopurinol was started at a dose of 50 mg/m^2^ per day with a max of 100 mg per day. Once allopurinol was added to the regimen, mercaptopurine was empirically dose reduced by at least 50% and adjusted based on ANC. ANC levels and labs were monitored by assessing each outpatient and inpatient complete blood count (CBC) beginning upon initiation of maintenance therapy through either completion of maintenance therapy or end of study period. The ANC value from the previous CBC was carried over until the next CBC was obtained to calculate time within ANC goal range of 500 to 1500 cells/microliter.

Hepatotoxicity, pancreatitis, and hypoglycemia were defined with the following criteria; AST > 160 unit/L/ ALT > 165 unit/L/ total bilirubin > 1.8 mg/L (CTCAE Grade 3 or greater), lipase > 90 unit/L, and blood glucose < 55 mg/dL (CTCAE Grade 2 or greater) respectively.^12^ Thresholds were less aggressive then contemporary protocol recommendations for therapy interruption due to hepatotoxicity in order to proactively identify patients with a higher likelihood of progressing to clinically relevant toxicity. These lab values were compared prior to and after allopurinol initiation to determine improvement of toxicity. Of note, lipase levels and blood glucose were only evaluated in symptomatic patients. 6‐MMPN and 6‐TGN levels were collected, as well as their ratio, however a specific goal was not defined. The daily doses of allopurinol and mercaptopurine, and weekly methotrexate administered were assessed. All adverse effects of allopurinol were recorded.

Statistical analysis was completed using Microsoft Excel. The primary endpoint was time within goal ANC prior to and after initiation of allopurinol. Secondary endpoints included; improvement in selective toxicities (hepatotoxicity, pancreatitis, and hypoglycemia) and 6‐MMPN to 6‐TGN ratio prior to and after allopurinol initiation. In addition, an exploratory endpoint assessing mercaptopurine daily dose reduction prior to and after allopurinol initiation was included.

## 3 RESULTS

Sixteen patients met inclusion criteria and 15 (94%) of which were included in this study. Exclusion was due to incomplete documentation. Pertinent patient demographics are shown in Table 1. Median patient age was 11.3 years of age (Range 1 to 23 years of age) with the most common diagnosis being B‐cell ALL (66%). At the time of data collection, 10 patients had completed maintenance therapy while 5 remained on active therapy. Median percent of maintenance days within goal ANC prior to and after initiation of allopurinol was 27.8 (IQR 22.6–44.9) and 41.6 (IQR 20.2–58.2) respectively (Table 2). All patients experienced selective toxicities; 15 (100%) hepatotoxicity, 1 (7%) pancreatitis, and 3 (20%) hypoglycemia. Improvement of toxicities was seen in 13/15 (87%), 1/1 (100%), and 2/3 (67%) respectively. Of note, the patient with pancreatitis was experiencing intermittent abdominal pain since receiving their final dose of pegaspargase approximately 1 month prior to starting Maintenance. This was initially attributed to gastritis, but when a serum lipase was checked after the first Maintenance cycle, it remained at least mildly elevated until starting allopurinol. Average 6‐MMPN:6‐TGN ratio prior to allopurinol initiation was 304:1 and after, allopurinol initiation improved to 15:1, resulting in a 95% reduction. Average mercaptopurine dose prior to and after allopurinol initiation decreased by about 56% (63 to 28 mg/m^2^/day). No allopurinol adverse effects were reported. No patients relapsed during the study period.

**TABLE 1:** Patient demographics.

Demographics	Median {*R*} or *N* (%) or median [IQR]
Age at diagnosis (years)	11.3 {1–23}
Start of maintenance therapy to allopurinol initiation (days)	217.0 [177.5–319.5]
Allopurinol dose (mg/m^2^/day)	50.0 [50.0–62.5]
Methotrexate dose (mg/m^2^/week)	Prior to allopurinol 17.8 [14.1–19.2] After allopurinol 17.6 [13.4–20.4]
Completed maintenance therapy	10.0 (66.7)
Race	Caucasian 12.0 (80.0) Asian 2.0 (13.3) Other 1.0 (6.7)
Ethnicity	Hispanic, Latino, or Spanish Origin 1.0 (6.7)
Diagnosis	B‐cell ALL 10 (66.6) Infant B‐cell ALL 1 (6.7) T‐cell ALL 3 (20.0) T‐cell Lly 1 (6.7)
Children's Oncology Group Treatment Protocol	AALL1131 9 (60.0) AALL0434 4 (26.6) AALL15P1 1 (6.7) AALL0232 1 (6.7)

Abbreviations: ALL, acute lymphoblastic leukemia; IQR, interquartile range; Lly, acute lymphoblastic lymphoma; *N* (%), sample size (percentage); *R*, range.

**TABLE 2:** Percent of time within goal absolute neutrophil count.

Absolute neutrophil count	Prior to allopurinol initiation median [IQR]	After allopurinol initiation median [IQR]
Below goal	0.0 [0.0–3.9]	1.7 [0.0–4.4]
At goal	27.8 [22.6–44.9]	41.6 [20.2–58.2]
Above goal	65.1 [51.1–76.4]	51.6 [30.3–78.0]

Abbreviation: IQR, interquartile range.

## 4 DISCUSSION

Results suggest that the use of allopurinol in pediatric patients with ALL and LLy receiving mercaptopurine during maintenance therapy is both safe and effective. Five patients were still receiving maintenance therapy, so we were not able to see the full effects of allopurinol addition however, our study supports the existing literature that addition of allopurinol to mercaptopurine maintenance regimens may resolve skewed metabolism and reduce hepatotoxicity. Allopurinol was shown to increase median time within goal ANC, improve selective toxicities, reduce 6‐MMPN:6‐TGN ratio, and reduce daily mercaptopurine dose without adverse effects. No patients included in our study experienced relapse during the study period. Limitations of this study include its retrospective, single center design. In addition, it did not compare patients receiving this intervention to a control group limiting the ability to identify patients that may have improved without intervention. Adherence was measured by family report only. Further studies are needed to identify patients who might benefit from early initiation of allopurinol and to help predict an optimal mercaptopurine dose reduction upon initiation of allopurinol.

## AUTHOR CONTRIBUTIONS

**Tecca Barone:** Conceptualization (equal); data curation (lead); formal analysis (lead); methodology (equal); visualization (lead); writing – original draft (lead); writing – review and editing (equal). **Smita Dandekar:** Conceptualization (equal); methodology (equal); supervision (equal); writing – review and editing (equal). **Daniel McKeone:** Conceptualization (equal); methodology (equal); supervision (equal); writing – review and editing (equal). **Kevin Mulieri:** Conceptualization (lead); data curation (supporting); formal analysis (equal); methodology (equal); supervision (lead); writing – review and editing (equal).

## CONFLICTS OF INTEREST STATEMENT

The authors have stated explicitly that there are no conflicts of interest in connection with this article.

## ETHICS STATEMENT

This study received IRB approval as exempt research.

## Data Availability

The authors had full access to all the data in the study and take responsibility for the integrity and accuracy of the data analysis.

## References

[1] American Cancer Society . Survival rates for childhood leukemias. American Cancer Society. Published 2019, Feb 12. Accessed June 13, 2023. https://www.cancer.org/cancer/types/leukemia-in-children/detection-diagnosis-staging/survival-rates.html

[2] Schmiegelow K , Nielsen SN , Frandsen TL , Nersting J . Mercaptopurine/methotrexate maintenance therapy of childhood acute lymphoblastic leukemia: clinical facts and fiction. J Pediatr Hematol Oncol. 2014;36(7):503‐517. 24936744 10.1097/MPH.0000000000000206 PMC4222610

[3] Bhatia S , Landier W , Shangguan M , et al. Nonadherence to oral mercaptopurine and risk of relapse in Hispanic and non‐Hispanic white children with acute lymphoblastic leukemia: a report for the Children's Oncology Group. J Clin Oncol. 2012;30:2094‐2101. 22564992 10.1200/JCO.2011.38.9924 PMC3601449

[4] Zaza G , Cheok M , Krynetskaia N , et al. Thiopurine pathway. Pharmacogenet Genom. 2010;20(9):573‐574. 10.1097/FPC.0b013e328334338f PMC3098750 19952870

[5] Wolters Kluwer . Mercaptopurine. Lexicomp. Updated June 13, 2023. Accessed April 10, 2023. http://online.lexi.com

[6] Seinen ML , van Asseldonk DP , Boer NK , et al. The effect of allopurinol and low‐dose thiopurine combination therapy on the activity of three pivotal thiopurine metabolizing enzymes: results from a prospective pharmacological study. J Crohns Colitis. 2013;7:812‐819. 23317929 10.1016/j.crohns.2012.12.006

[7] Witte TN , Ginsberg AL . Use of allopurinol with low‐dose 6‐mercaptopurine in inflammatory bowel disease to achieve optimal active metabolite levels: a review of four cases and the literature. Can J Gastroenterol. 2008;22(2):181‐185. 18299738 10.1155/2008/870981 PMC2659140

[8] Brackett J , Schafer ES , Leung DH , Bernhardt MB . Use of allopurinol in children with acute lymphoblastic leukemia to reduce skewed thiopurine metabolism. Pediatr Blood Cancer. 2014;61(6):1114‐1117. 24376133 10.1002/pbc.24913

[9] Stuckert AJ , Schafer ES , Bernhardt MB , Baxter P , Brackett J . Use of allopurinol to reduce hepatotoxicity from 6‐mercaptopurine (mercaptopurine) in patients with acute lymphoblastic leukemia (ALL). Leuk Lymphoma. 2020;61(5):1246‐1249. 31842647 10.1080/10428194.2019.1702183

[10] Giamanco NM , Cunningham BS , Klein LS , Parekh DS , Warwick AB , Lieuw K . Allopurinol use during maintenance therapy for acute lymphoblastic leukemia avoids mercaptopurine‐related hepatotoxicity. J Pediatr Hematol Oncol. 2016;38(2):147‐151. 26808368 10.1097/MPH.0000000000000499

[11] PennState Health Children's Hospital . Facts and statistics. PennState Health. Published 2023. Accessed Apr 10, 2023.

[12] U.S. Department of Health and Human Services . Common Terminology Criteria for Adverse Events (CTCAE). Cancer. Published 2017, Nov 17. Accessed May 5, 2023.

